# Transoral Laser Microsurgery Outcomes in Early-Stage Glottic Laryngeal Cancer at a Tertiary Center

**DOI:** 10.7759/cureus.102279

**Published:** 2026-01-25

**Authors:** Deniz Baklacı, Duygu Erdem, Mustafa Dalgic, Oguz Can Karakas, Hatice Cakirlar, Huseyin Isik, Ergin Bilgin

**Affiliations:** 1 Otolaryngology - Head and Neck Surgery, Zonguldak Bülent Ecevit University, Zonguldak, TUR; 2 Otolaryngology - Head and Neck Surgery, Zonguldak Atatürk State Hospital, Zonguldak, TUR

**Keywords:** cordectomy, diode laser, glottic cancer, laryngeal neoplasms, survival

## Abstract

Introduction and aim: Curative treatment options for early-stage glottic laryngeal cancer include primary radiation therapy, open surgical techniques, and transoral laser microsurgery. In the last decade, transoral laser microsurgery has become a standard approach in many institutions due to its minimally invasive nature. This study aimed to evaluate the oncologic outcomes, complication rates, and therapeutic efficacy of transoral diode laser microsurgery in patients with early-stage (Tis, T1, T2) glottic laryngeal cancer.

Materials and methods: The retrospective data of 52 patients treated with transoral diode laser microsurgery at the ENT Clinic of Zonguldak Bülent Ecevit University between January 2021 and December 2024 were analyzed. Patients' demographic characteristics, tumor stages, surgical margin status, postoperative complications, recurrence rates, and survival outcomes were evaluated.

Results: Of the 52 patients in the study, 25 were in Tis, 20 in T1, and seven in T2 stage. The mean age of the patients was 65.5 years; 94.2% were male, and 5.8% were female. The mean follow-up period was 25.3 months. The postoperative complication rate was 8%, and the most common complications were vocal cord edema and granuloma formation. The local recurrence rate was 9.6%. The recurrence rate was higher in patients with anterior commissure involvement. No patient required a tracheotomy.

Conclusion: Transoral diode laser microsurgery is an effective treatment modality for early-stage glottic laryngeal cancers with a low complication rate and short hospitalization period. The risk of recurrence is higher, especially in patients with anterior commissure involvement, and this patient group should be followed more closely.

## Introduction

Laryngeal cancers are among the most common malignancies of the head and neck region and represent a significant global health problem [1,2]. Early-stage glottic cancer includes carcinoma in situ (Tis) as well as T1 and T2 tumors of the vocal cords without cervical lymph node metastasis [1].

The primary treatment modalities for early-stage glottic carcinoma are transoral laser microsurgery, open surgical procedures, and radiotherapy [3-6]. In addition to these, transoral microsurgical excision using cold instruments (cold knife technique) remains a viable and widely used option, particularly for localized lesions where preserving the layered structure of the vocal fold is paramount. Among these, laser surgery has gained wide popularity due to its advantages, including excellent hemostasis and surgical precision, minimal tissue excision, shorter operative times, reduced postoperative pain, and shorter hospital stays [7-10].

The use of the CO_2_ laser in laryngeal surgery began in the 1970s when Strong and Jako first reported its application [11]. Since then, it has demonstrated several benefits, such as enabling bloodless surgery, reducing or eliminating the need for tracheotomy, and minimizing postoperative edema. However, limitations of CO_2_ laser surgery include the requirement for bulky equipment and technical challenges in cases with anterior commissure involvement or subglottic extension [12].

Diode laser microsurgery has emerged as an alternative with distinct advantages. Its portable and flexible design facilitates ease of use, while excision is achieved by delivering energy to the surgical field in single or continuous pulses via fiberoptic cables [3,7,12-14]. Typically used at wavelengths between 805 and 980 nm, the diode laser also provides superior accessibility through an angled fiberoptic guide tip, which can reach anatomical areas that are difficult to access with the CO_2_ laser [12,14]. The present study evaluates the complications and early oncological outcomes of 52 patients with Tis, T1, and T2 glottic carcinoma who underwent diode laser microsurgery between January 2021 and December 2024.

## Materials and methods

Fifty-two patients diagnosed with Tis, T1, and T2 glottic squamous cell carcinoma of the larynx who accepted surgery were operated on with endolaryngeal diode laser microsurgery in the Department of Otolaryngology, Bülent Ecevit University Hospital.

Inclusion criteria

Patients were included in the study if they met the following criteria: (1) histologically confirmed squamous cell carcinoma of the glottic larynx, (2) clinical stage Tis, T1a, T1b, or T2 according to the American Joint Committee on Cancer (AJCC)/Union for International Cancer Control (UICC) tumor, node, metastasis (TNM) classification system, (3) primary treatment with transoral diode laser microsurgery at our institution between January 2021 and December 2024, (4) adequate visualization of the tumor during direct laryngoscopy, and (5) a minimum follow-up period of six months.

Exclusion criteria

Patients were excluded based on the following criteria: (1) non-squamous cell malignancies of the larynx, (2) advanced-stage glottic cancer (T3 or T4), (3) presence of cervical lymph node or distant metastasis at the time of diagnosis, (4) history of previous laryngeal surgery, radiotherapy, or chemotherapy for head and neck cancer, (5) inadequate endoscopic exposure of the larynx (e.g., due to anatomical limitations), and (6) incomplete medical records or lack of follow-up data.

Tumor staging was performed using the AJCC/UICC TNM classification system. Cordectomy procedures were classified according to the European Laryngological Society (ELS) classification system, which defines the extent of resection based on the anatomical depth and breadth of the surgical excision as follows: type I (subepithelial cordectomy) - resection of the vocal fold epithelium, passing through the superficial layer of the lamina propria; type II (subligamental cordectomy) - resection of the epithelium, Reinke’s space, and the vocal ligament; type III (transmuscular cordectomy) - resection through the vocalis muscle; type IV (total cordectomy) - complete resection of the vocal fold, extending from the thyroid cartilage to the muscular process of the arytenoid; type V (extended cordectomy) - resection that extends beyond the vocal fold to include the contralateral vocal fold (Va), the arytenoid (Vb), the ventricular fold (Vc), or the subglottis (Vd) [10]. Both of these tools are open access, widely accepted in clinical practice, and do not require any licensing for academic use.

Disease-free survival (DFS) was defined as the time from the date of surgery to the first documented local or regional recurrence, or to the last follow-up visit for patients without recurrence. Patients who died without evidence of recurrence or who were lost to follow-up were censored at the date of last contact. Survival probabilities were estimated using the Kaplan-Meier method, and curves were stratified by tumor stage (Tis, T1, T2) to allow comparison of recurrence-free outcomes across groups.

All patients were routinely examined with a 70° rigid or flexible endoscope in the outpatient clinic in the preoperative period. Then, direct laryngeal biopsies were taken under general anesthesia in patients with suspected malignancy. Patients whose biopsy results were reported as squamous cell carcinoma were clinically evaluated for lymph node and distant metastasis with contrast-enhanced neck and thorax CT in the preoperative period. Direct laryngeal examination and CT findings were evaluated to determine TNM classification.

Biopsy results and treatment options were explained to all patients in detail. Treatment was determined according to the patient's choice and easy visualization of the tumor on direct laryngoscopy. Preoperative anesthesia preparations were initiated, and consent was obtained for patients whose tumor was easily visible on direct laryngoscopy and whose treatment preference was diode laser surgery. All patients were routinely hospitalized one day before surgery. A diode laser (Gigaa VelasII-30B; Hubei, China: Wuhan Gigaa Optronics Technology Co. Ltd.) with the following characteristics was used to remove the tumor: power 6-10 W, wavelength 980 nm, fiber 400 µm, and continuous wave mode.

**Figure 1:**
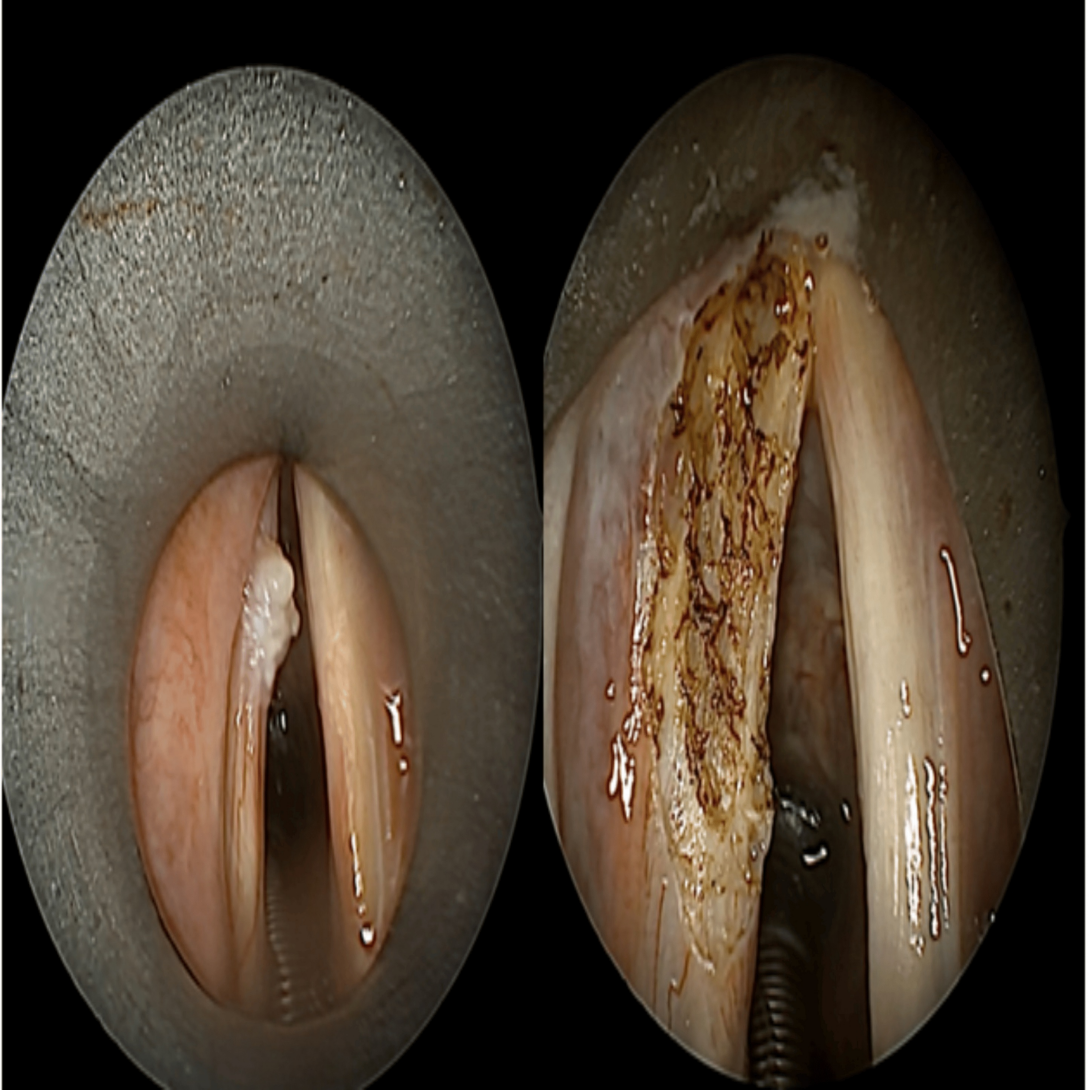
Preoperative (left) and postoperative (right) view of diode laser surgery procedure (original clinical image from our institution).

Patients were intubated with a protected endotracheal tube specially designed for the laser. The proximal part of this double-cuffed tube was inflated with 3 cc of saline, and the distal part with 7 cc of saline. The patient was placed in a supine position with the head fully tilted back. A rigid laryngoscope was used to visualize the vocal cords through the laryngoscope. Once an adequate field was provided, the laryngoscope was secured with a sling arm. The microscope was then positioned. Tumors were excised using the continuous pulse mode of the diode laser (Figure 1).

After tumor removal, mapping was performed, and samples were sent for frozen examination from all surgical margins. The operation was completed in patients with negative frozen results. Patients with positive results underwent additional resection until the results were negative. After negative results were obtained, hemostasis was achieved in the operating field, and the surgical field was left for secondary healing. Cold steam therapy was applied to the patients in the early postoperative period. Seven-day antibiotherapy and antireflux therapy were initiated at discharge. Absolute sound rest and an antireflux diet were recommended. The surgical field was observed endoscopically during the first week of the outpatient clinic visit.

Patients who had undergone laser cordectomy were examined with a 70° rigid endoscope or flexible endoscope at three-month intervals in the first postoperative year and every six months in the second year in the outpatient clinic. Patients without recurrence were followed up annually after two years. Biopsy was repeated in patients with recurrence.

## Results

In our clinic, 52 patients underwent surgery, of whom 25 were diagnosed with Tis, 20 with T1, and seven with T2 stage glottic laryngeal carcinoma. Forty-nine patients were male, and three were female. The ages of the patients ranged from 44 to 84 years, with a mean age of 65.5 years. The mean follow-up period was 25.3 months (range: 6-50 months). Patients with shorter follow-up durations were those who had undergone surgery more recently. No perioperative mortality was observed in any patient. The demographic and clinical characteristics of the 52 patients are summarized in Table 1. Cordectomies were classified according to the European Laryngological Society (ELS) classification system [10]. The types of cordectomy performed in these patients are presented in Table 2 [10].

**Table 1:** Demographic and clinical characteristics of the study population. TNM: tumor, node, metastasis

Characteristics		Value
Total number of patients		52
Age (years)	Mean	65.5
	SD	9.2
	Range	44-84
Gender, n (%)	Male	49 (94.2)
	Female	3 (5.8)
Smoking history, n (%)	Smoker	50 (96.2)
	Non-smoker	2 (3.8)
Smoking exposure, pack-years	Mean	28
	SD	10.5
	Range	5-40
Tumor stage, n (%)	TNM - Tis	25 (48.1)
	TNM - T1	20 (38.5)
	TNM - T2	7 (13.5)
Anterior commissure involvement, n (%)		12 (23.1)
Follow-up duration, months	Mean	25.3
	SD	12.4
	Range	6-50
Hospitalization duration, days	Mean	1.63
	SD	0.8
	Range	1-5

**Table 2:** Types of laser cordectomy in patients with Tis, T1, and T2 stage glottic laryngeal carcinoma. Distribution of laser cordectomy types in patients with Tis, T1, and T2 stage glottic laryngeal carcinoma. Cordectomy types were classified according to the ELS classification. TNM: tumor, node, metastasis; ELS: European Laryngological Society

Laser type/TNM	Type I	Type II	Type III	Type IV	Type V
Tis	6	14	5	0	0
T1	4	4	12	0	0
T2	0	0	2	2	3

The duration of hospitalization ranged from one to five days, with a mean of 1.63 days. Two patients had no history of smoking, while the duration of smoking among the other patients ranged from five to 40 years, with a mean of 28 pack-years. No significant complications were observed during the perioperative period. No patient developed laryngeal stenosis postoperatively, and tracheotomy was not required in any case.

During postoperative follow-up, a total of five patients (9.6%) experienced recurrence, of whom three patients (5.8%) had local recurrence. The stages of local recurrence were one Tis and two T1. Local recurrences were detected at six, nine, and 10 months postoperatively. Two patients underwent repeat laser cordectomy, while one patient refused reoperation and was referred for radiotherapy. Two patients with T2-stage disease developed regional recurrence, were deemed inoperable, and were scheduled for chemoradiotherapy. Two patients initially staged as T2 before surgery progressed to T4 during follow-up. One patient who underwent chemoradiotherapy for regional recurrence after laser surgery died at 20 months of follow-up due to secondary causes. Additionally, two patients who underwent laser cordectomy required repeat laser cordectomy due to close surgical margins, and no recurrence was observed during follow-up.

In the postoperative period, four patients (8%) developed vocal cord edema, which resolved with medical treatment. Two patients (4%) developed granulomas; one regressed spontaneously during follow-up, while the other required surgical excision. Furthermore, two patients (4%) developed anterior glottic webs; however, no surgical intervention was required, and they were followed closely.

Among the 52 patients who underwent diode laser cordectomy, 12 patients (23%) had anterior commissure involvement. Of these, four were Tis, six were T1, and two were T2 stage. Postoperatively, synechiae were observed in three patients, and a web was noted in one patient during follow-up. Recurrence occurred in three patients (25%) with anterior commissure involvement. One of these underwent repeat laser cordectomy, while the remaining two were staged as T4 and referred for chemoradiotherapy.

**Figure 2:**
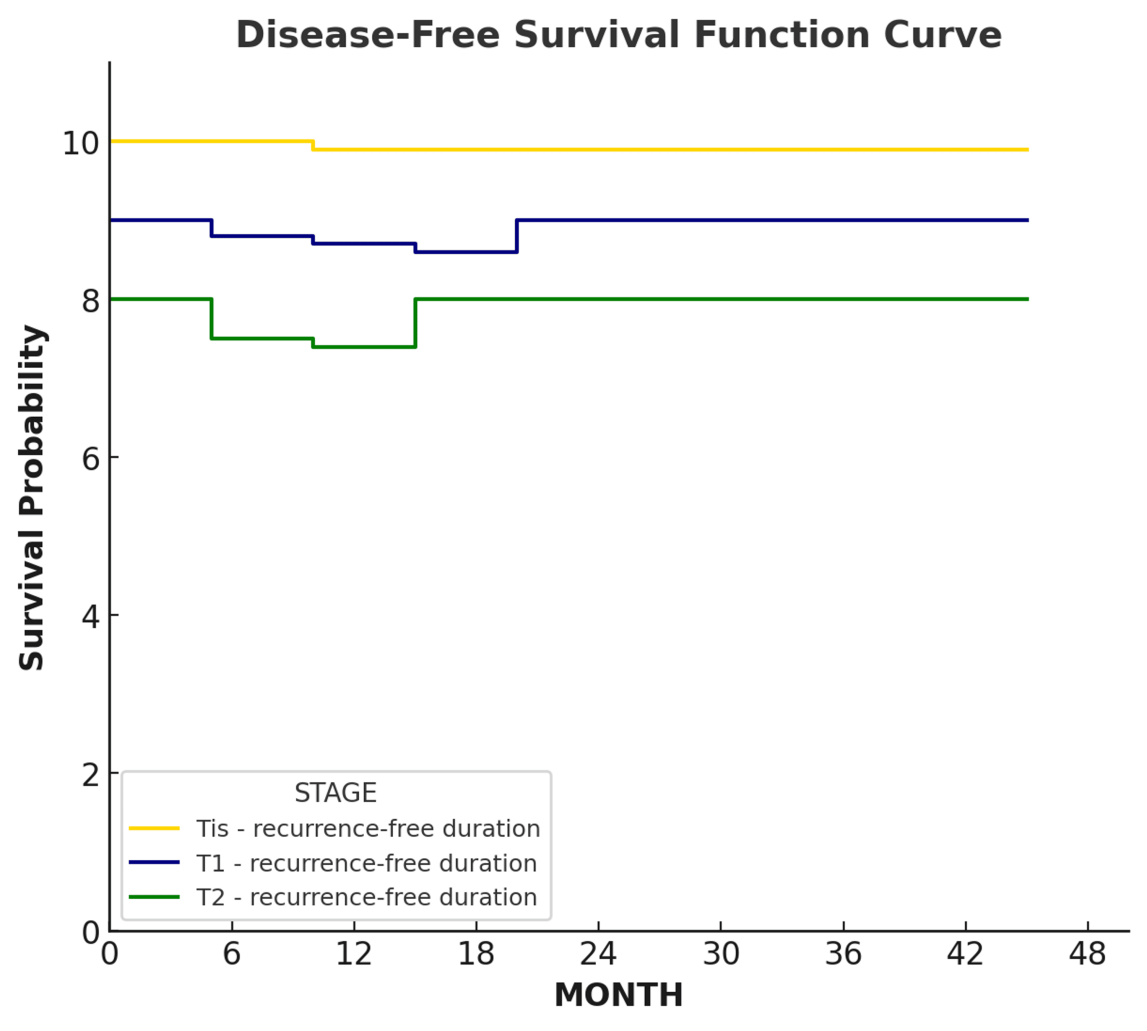
Disease-free survival curve. Disease-free survival curve according to tumor stage (Tis, T1, T2) based on the AJCC/UICC TNM classification system. AJCC/UICC: American Joint Committee on Cancer/Union for International Cancer Control; TNM: tumor, node, metastasis

According to Kaplan-Meier analysis, disease-free survival (DFS) rate was evaluated in patients with early-stage glottic laryngeal carcinoma (Tis, T1, T2) (Figure 2) [10]. A total of 52 patients were divided into the following three groups according to tumor stage: Tis (25 patients), T1 (20 patients), and T2 (seven patients). In the Tis group, recurrence was observed in only one patient (5%), and the disease-free survival rate was 95%. In T1-stage laryngeal cancer, recurrence occurred in two patients (8%), yielding a disease-free survival rate of 92%. In the T2 group, recurrence was seen in two patients (28.6%), and the disease-free survival rate was 71.4%. Overall, five recurrence cases (9.6%) were observed, and the disease-free survival rate was 90.4%. These findings indicate a high rate of recurrence-free survival, particularly in the Tis group and overall in the study population. The recurrence rate was higher in T2-stage laryngeal carcinoma compared with other stages. The overall survival rate was calculated as 98.1%.

## Discussion

Among the treatment options for early-stage glottic laryngeal cancer are open partial surgery, radiotherapy, and transoral laser surgery. Transoral microsurgery can be performed using either laser systems or cold instruments. Cold instrument microsurgery is often preferred for its ability to provide a high-quality specimen for histopathological margin assessment without thermal artifact and for its preservation of the vocal fold's fine architecture. However, it may be limited by intraoperative bleeding in more extensive resections. Open partial surgery requires prolonged hospitalization due to postoperative pain, edema, wound infection, and the need for tracheotomy. Radiotherapy, on the other hand, has disadvantages, such as prolonged treatment duration and mucosal damage [8].

Diode laser is a recently introduced method for the treatment of glottic tumors. It is small, portable, and flexible, and it performs excision by delivering energy to the surgical field in single or continuous pulses. It is suitable for both endoscopic and microscopic use [3,7,12-14]. The angled fiber-optic tip allows access to areas difficult to reach with the CO_2_ laser [12]. The treatment of laryngeal tumors originating from or involving the anterior commissure with CO_2_ laser is challenging due to the anatomical characteristics of the region, the difficulty of exposing the tumor, and the technical limitations of vertical laser application [15]. In a study by Blanch et al., the complication rate of CO_2_ laser surgery for anterior commissure tumors was found to be 14% [15].

Diode lasers have several advantages over conventional solid-state and gas lasers (such as neodymium-doped yttrium aluminum garnet {Nd:YAG}, potassium titanyl phosphate/yttrium aluminum garnet {KTP/YAG}, argon), which are bulky, difficult to transport, require water cooling, have longer warm-up times, and need regular maintenance [9]. The magnified microscopic view and the precision of the diode laser beam allow resection in a narrow area, thus preserving functional paraglottic tissue, including the vocalis muscle, in early-stage glottic tumors. The 980 nm diode laser is a new technology that has been introduced recently, with the main difference from the 810 nm type lying in their therapeutic effects. Treatment with the 980 nm diode laser has been shown to accelerate wound healing by altering the expression of PDGF and bFGF genes, which are responsible for cell proliferation and fibroblast growth stimulation [10].

The need for tracheotomy in laser procedures is extremely low and has been reported between 0% and 6% in previous studies [16]. In our study, no patient required a tracheotomy. Endotracheal tube ignition can cause significant morbidity and mortality during laser surgery [17]. By using a suitable tube for laser surgery and covering the tube cuff with wet pads, no complications occurred in the present study. Of the 52 patients included, five (9.6%) experienced recurrence, three were local recurrences, and two were regional recurrences. Recurrence rates after diode laser use range from 6.2% to 11.1%, and the recurrence rate in the present study was consistent with reported rates [2,4,7,8].

Tunçel and Cömert reported that all recurrences occurred in T1b (one patient) and T2 (three patients) tumors, and that all of them had positive postoperative margins [4]. In the study by Ferri and Armato, all local recurrences occurred in T1b tumors and were localized to the anterior commissure [8]. Similarly, in our study, three of the five recurrences involved the anterior commissure. Of these three patients, two had T2 glottic carcinoma, and one had T1 glottic carcinoma. A review of the literature reveals that recurrence rates after CO_2_ laser use range from 2.9% to 25.9%, and it is noteworthy that recurrence rates in CO_2_ laser surgery vary widely [18-23]. Positive surgical margins after CO_2_ laser surgery range from 3.7% to 16.3% [18-21,24].

As with the diode laser, complication rates in CO_2_ laser surgery are rare or absent, with bleeding being the most common complication [18,19]. In the study by Motta et al., which included 790 patients treated with CO_2_ laser, emphysema due to cricothyroid penetration developed, and 36 complications (5%) were observed. Four patients required a tracheotomy [19]. There are a few studies in the current literature focusing on complications. In the study by Ellies and Steiner, which included 1,528 patients undergoing laser surgery, bleeding was the most common postoperative complication and was suggested to be associated with larger tumor size and wider wound defects [25]. Nevertheless, although rare, laryngeal stenosis and aspiration pneumonia may develop.

Ellies and Steiner reported perichondritis in three patients and marked skin emphysema due to cartilage defects in four patients. A pressurized dressing was recommended for the patient with emphysema, and clindamycin, absorbed by cartilage, was suggested as treatment for perichondritis [25]. In a study of 64 patients conducted by Tunçel and Cömert, early postoperative complications such as hemorrhage, abscess formation, and perichondritis were reported; however, none of these were observed in the present study [4]. In the study by Edizer and Cansız, among 13 patients with anterior commissure involvement treated with transoral laser surgery for T1, T2, and T3 stage laryngeal cancer, granuloma and synechia formation occurred in eight patients [26]. In the present study, synechia was observed in four of 12 patients with anterior commissure involvement. In the study by Edizer and Cansız, the local recurrence rate of tumors involving the anterior commissure was reported as 15%, whereas in the present study, this rate was 25%.

In our series, the disease-free survival rate after diode laser surgery was 90.4% at four years, consistent with previous reports of diode laser surgery in early-stage glottic cancer [3,4,8,12,13]. As in our study, retrospective analyses conducted in Türkiye have demonstrated the importance of transoral laser surgery in the treatment of early-stage glottic cancer [27].

Since a CO_2_ laser device was not available in our clinic during the study period, a direct comparison with a CO_2_ laser could not be made. Therefore, instead of a head-to-head comparison, we aimed to evaluate the oncologic outcomes of diode laser surgery and compare our findings with existing studies using diode laser systems in the literature. This approach allowed us to assess the efficacy and safety of diode laser surgery in the treatment of early-stage glottic carcinoma based on comparable methodologies.

This study has several limitations that should be considered when interpreting the results. First, its retrospective, single-center design is inherently subject to selection bias and potential inconsistencies in documentation. Second, the sample size was relatively modest, and the follow-up duration was heterogeneous, which may limit the robustness of long-term oncologic outcomes, particularly for the smaller T2-stage subgroup. Third, the study lacked a control group (e.g., CO_2_ laser or radiotherapy), meaning our findings represent descriptive institutional outcomes rather than a comparative analysis of efficacy. Fourth, due to the retrospective nature of the data, standardized functional outcome measures, such as the Voice Handicap Index (VHI), grade, roughness, breathiness, asthenia, strain (GRBAS) scale, or formal phoniatric evaluations, were not systematically available and could not be evaluated. Finally, the results may be influenced by institution-specific factors, including surgeon experience and the specific diode laser settings used. Future prospective multicenter studies with longer follow-up and validated functional assessment tools are necessary to more comprehensively evaluate the role of diode laser microsurgery in early-stage glottic cancer.

## Conclusions

This retrospective study evaluated the four-year clinical outcomes of patients with laryngeal cancer treated with diode laser surgery at Tis, T1, and T2 stages. Our study shows that diode laser surgery is an effective and safe option in treating early-stage laryngeal cancer. In most treated patients, local control achieved low recurrence rates and satisfactory overall survival rates. In addition, since laser surgery is a minimally invasive approach, patients had short postoperative recovery times and reduced hospitalization. However, it is important to consider the patient's general condition, tumor characteristics, and surgical requirements in the treatment choice. Future studies suggest that this treatment modality should be evaluated in more detail in larger patient groups and with longer follow-ups.
